# AI driven mechanical circulatory support: Can AI and Impella team up to beat cardiogenic shock

**DOI:** 10.1002/ehf2.14995

**Published:** 2024-07-24

**Authors:** Robert Zymliński, Wojciech Tokarczyk, Szymon Urban

**Affiliations:** ^1^ Institute of Heart Diseases, University Clinical Hospital in Wroclaw Wroclaw Medical University Wroclaw Poland; ^2^ Institute of Heart Diseases University Clinical Hospital in Wroclaw Wroclaw Poland

Temporary mechanical circulatory support (MCS) has recently become indispensable in the treatment of cardiogenic shock and serves as a crucial backup for high‐risk percutaneous coronary interventions (PCI).^1^

However, the crucial elements leading to success—an adequate patient selection, timing of implantation, central haemodynamic assessment, flow adjustment, anticoagulation management, and post‐implantation period—still remain unclear. The efficacy of MCS has been demonstrated in the first randomized controlled trial, known as the ‘DANGER Shock’ trial.^2^ Among the most commonly used devices, the Impella not only provides mechanical circulatory support and cardiac unloading^3^ but also functions as an integrated monitoring system. It gathers comprehensive hemodynamic data, including pressures, cardiac output, left ventricular systolic and end‐diastolic pressure, cardiac power output, and pulmonary artery and central venous pressures supported by detailed patients clinical assessment. This robust data environment due to their complexity and heterogeneity creates an ideal setting for the implementation of artificial intelligence (AI) techniques.

Modern medicine and technological advancements equip us with comprehensive tools for phenotyping patients admitted to hospitals with cardiogenic shock. This facilitates the implementation of personalized treatment strategies derived from data‐driven methods such as machine learning, clustering, and other artificial intelligence‐based technologies. Employing these approaches can enhance patient outcomes and the data acquired can be pivotal in designing new clinical trials. These trials, in turn, can lead to the refinement and advancement of therapeutic protocols.^4^

In their article, Consolo et al. explore the future directions of patient management with MCS using AI, highlighting several intriguing avenues for advancement.^5^ One noteworthy development is the Impella Smart Assist system by Abiomed. This system represents the first attempt to consolidate data from the Impella device for various clinical applications, including motor adjustment, timely de‐escalation, weaning, and system positioning assessment. Despite the promising potential of these applications, their clinical impact remains largely experimental, and substantial advancements in patient management have yet to be realized.

While the efforts to utilize Impella‐derived data for treatment management are commendable, they are currently insufficient. Effective AI‐driven clinical decision support requires a significantly broader dataset than what Impella alone can provide. Relevant data from serial blood gas analyses, laboratory results, and echocardiographic imaging,^6^, ^7^ all of which are available in numerical formats, must be integrated.^8^, ^9^ Only by incorporating a wider range of clinical, functional, and laboratory data can AI models address critical issues such as fluid therapy adjustment, renal injury risk assessment,^10^, ^11^ and optimization of catecholamine dosages.

One particularly exciting concept is the ‘digital twin’—a virtual clone of the patient that analyzes data from the predefined period of time of monitoring to evaluate potential therapeutic decisions. This initiative could broaden physicians' perspectives by providing insights into the consequences of various treatment choices without experimenting on the actual patient.^12^ However, the predictive capabilities of such a model are limited by the scope of data available to the algorithm. Even with the integration of additional clinical data, the dataset remains constrained to parameters that can be named and quantified, thus being less comprehensive than the information a physician uses. Consequently, the projections made by the model should be interpreted with caution.

The Impella Connect system, which gathers data from multiple centres and allows simultaneous monitoring of decision outcomes, presents a significant opportunity for self‐supervised machine learning (ML). In this context, the model observes the consequences of its decisions, learning and refining its predictions over time. This approach could greatly enhance the efficacy of AI‐driven clinical management.^13^, ^14^

AI can also address the risk assessment and management of renal injury associated with MCS.^15^ Haemolysis caused by microaxial flow poses a significant risk of permanent kidney damage, potentially necessitating chronic dialysis. The management of shock dynamics progressing to multiple organ failure (MOF) often eludes the capabilities of standard laboratory tests and clinical evaluations. Despite extensive research into the pathophysiology of shock yielding significant data, these insights frequently remain impractical for routine clinical application due to their nuanced changes. Advanced AI techniques can elucidate the relationships among parameters monitored in intensive care units, thereby offering clinicians more precise and actionable data to inform therapeutic interventions. Balancing the risk of renal injury with the efficiency of circulatory support is a complex issue that AI models could potentially solve. Such models would need to account for various factors, including creatinine levels, haptoglobin, bilirubin, blood gas measurements, urine output, fluid balance,^16^ and electrolyte management.^17^ Additionally, tracking the dynamics of these parameters over time is crucial. Our proposed application of AI in the optimization of LVAD therapy for the management of cardiogenic shock is delineated in *Figure* 1.

**Figure 1:**
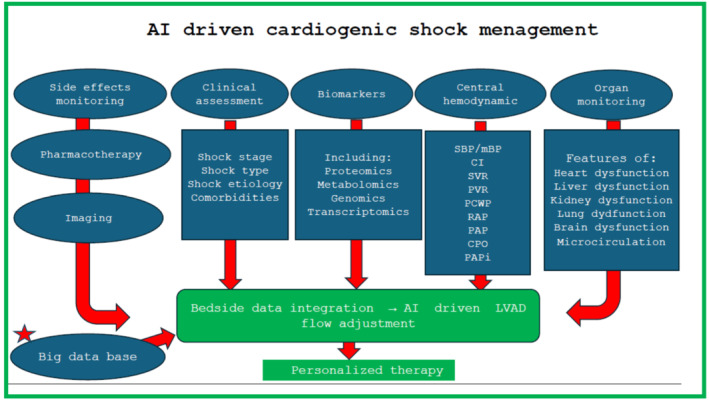
Proposal for the use of artificial intelligence optimizing the use of LVAD in cardiogenic shock management based on the integration of various data. BP, blood pressure; CI, cardiac index, CPO, cardiac power output = mean arterial pressure × cardiac output/451; mBP, mean blood pressure; PAP, pulmonary artery pressure; PAPi, pulmonary artery pulsatility index = systolic pulmonary arterial pressure − diastolic pulmonary arterial pressure/right atrial pressure; PVR, pulmonary vascular resistance; RAP, right atrial pressure; SVR, systemic vascular resistance.

In conclusion, the integration of AI in MCS management holds tremendous potential for improving patient outcomes. However, realizing this potential requires the consolidation of a broader range of clinical data and cautious interpretation of AI model predictions. The journey toward AI‐enhanced MCS is just beginning, and future advancements will depend on the continuous refinement of data integration and model accuracy. As we move forward, it is essential to balance technological innovations with clinical prudence to ensure the best possible care for patients.

## Funding

Not applicable.

## Conflict of interest

None declared.
